# Fermented Mare Milk and Its Microorganisms for Human Consumption and Health

**DOI:** 10.3390/foods13030493

**Published:** 2024-02-03

**Authors:** Francesca Martuzzi, Piero Franceschi, Paolo Formaggioni

**Affiliations:** 1 Department of Food and Drug Science, University of Parma, Via delle Scienze, 43124 Parma, Italy; francesca.martuzzi@unipr.it; 2 Department of Veterinary Science, University of Parma, Via del Taglio 10, 43126 Parma, Italy; paolo.formaggioni@unipr.it

**Keywords:** mare milk, koumiss, airag, lactic acid bacteria, yeasts

## Abstract

Mare milk is consumed by approximatively 30 million people in the world. In countries in Asia and East Europe, mare milk is mainly consumed as source of fermented products, called koumiss, airag or chigee, alcoholic beverages obtained by means of a culture of bacteria and lactose-fermenting yeasts. Recent research concerning mare milk and its derivatives deals mainly with their potential employment for human health. Studies about the isolation and characterization of *Lactobacillus* spp. and yeasts from koumiss have been aimed at assessing the potential functional properties of these micro-organisms and to find their employment for the industrial processing of mare milk. The aim of this literature review is to summarize recent research about microorganisms in fermented mare milk products and their potential functional properties.

## 1. Introduction

Mare milk is a fundamental aliment for the people of the Central Asia steppes. It is consumed also in Europe, in particular in Hungary and in the Netherlands. It was estimated that nearly 30 million people consume it regularly [1]. Besides the use of this milk as a source of valuable nutrients, since ancient times it has been considered as a sort of medication for its health-promoting characteristics in the regions of the former USSR and Western Asia. It has been extolled for its many healing properties in some papers, but clinical studies effectively proving its positive effects are scarce [2,3,4].

In the recent years of the 20th century, in Europe, studies about equine milk have mostly dealt with protein compounds—the identification and characterization of caseins and whey proteins (see reviews by Martuzzi and Doreau [5] and by Uniacke-Lowe et al. [6])—and to a lesser extent other milk components [7], with some interest for its possible use as a substitute for bovine milk for children with intolerance or allergy [8,9,10]. In particular, whereas most studies in the world have dealt with horse’s milk, in Italy interest is more focused on donkey’s milk, traditionally used in the past for orphaned or abandoned children, when formulas were not yet available [11,12,13]. An extensive review about equid milk (horse and donkey) composition and tolerability in human nutrition was published by Salimei and Fantuz [14].

Whereas milk from most domestic ruminants is widely consumed as cheese after processing, cheesemaking from equine milk is not possible, mainly due to its scarce casein content. No curd is formed on the addition of rennet; under acidic conditions only a weak coagulum appears, manufactured especially in the Netherlands for the production of yoghurt-type products with the addition of fruit extract [6,15].

Equine milk is rich in its lactose content, and since most populations in Asia present lactose malabsorption [16,17], in this part of the world mare milk undergoes fermentation before consumption, and the resulting products are called koumiss [18] or qymyz (Eastern Europe, former Soviet Union Republics) [19,20], airag (Mongolia) [21,22] or chigee (Inner Mongolia and Xinjiang, China) [23,24], which are alcoholic beverages obtained by means of a mixed culture of bacteria and yeasts. During fermentation, the lactose is converted into lactic acid, ethanol and carbon dioxide, and the milk becomes an accessible nutriment for lactose-intolerant people. In addition, acidifying fermentation is the oldest method of milk conservation [25]. Organic residue analysis, using δ^13^C and deuterium isotope (δD) values of fatty acids, has revealed processing of mare milk products in ancient potsherds dating back to about 3500 B.C.E., found in Northern Kazakhstan [26]. Kazakhstan is still at present the nation with the largest koumiss production in the world [27,28].

Nevertheless, until recently, little was known about the numerous studies carried out into koumiss in the former USSR, where this product has been consumed for centuries, due to the difficult accessibility of these papers, written in the Russian language. This lack of knowledge was overcome by a review by Kondybayev et al. [19] which surveyed many studies of soviet authors.

The reader can find a lot of data about the composition of and variability in mare milk in the aforementioned reviews. Recent research about mare milk deals mainly with its derivatives, in particular fermented milk products and the potential probiotic properties of the microorganisms living within [29].

The aim of this review is to present recent advancements in fermented mare milk research, with a particular focus on its potential functional properties and effects on human health.

The following search strategy for the review was adopted: to conduct the literature search and include relevant references, we scanned the databases Medline, PubMed, ScienceDirect, and CNKI for Chinese papers. For the search in the online databases, we used the following keywords: (koumiss), (kumiss), (airag), and (mare milk). In addition, since mare milk is one of the topics of our research group, we had collected most of the considered papers during the preceding years and therefore they were present in our collection of studies already. Regarding the recent literature, at the first screening process, we scanned titles and abstracts of the yielded articles or book chapters. Afterwards, if the publication did not show any signs of incoherence, we screened the full text and searched the sections “similar articles” or “related documents” according to the diverse databases. Three papers were excluded as they studied several traditional fermented foods obtained by the milk of diverse species (such as yak, cow, and camel) together, and it was not possible to select data regarding the specific product obtained by mare milk.

## 2. Microorganisms in Fermented Mare Milk Products

The most abundant microorganisms naturally present in milk can be classified in order of their possible roles (i) microorganisms involved in dairy fermentation (e.g., *Lactococcus*, *Lactobacillus*, *Streptococcus*, *Propionibacterium* and fungal populations); (ii) involved in spoilage (e.g., *Pseudomonas*, *Clostridium*, *Bacillus*, and other spore-forming bacteria); (iii) involved in food disease (e.g., *Listeria*, *Salmonella*, *Escherichia coli*, *Campylobacter* and mycotoxin-producing fungi); and (iv) involved in promoting health (e.g., lactobacilli and bifidobacteria) [30]. Human milk from healthy women contains up to 10^9^ microbes L^−1^ [31]. These organisms come mainly from the nipple and surrounding skin [31]. Ward et al. studied the complexity of the bacterial community in human milk, finding over 360 prokaryotic genera, mainly belonging to the phyla of Proteobacteria (65%) and Firmicutes (34%), and the *genera* of *Pseudomonas* (61.1%), *Staphylococcus* (33.4%) and *Streptococcus* (0.5%) [32]. Microbial colonization during the first few weeks of life in the gastrointestinal tracts of humans and farm animals is remarkably similar. Bifidobacteria are the predominant lactic acid bacteria (LAB) in infants, whereas lactobacilli are the primary LAB in the tract of new-born foals [33].

The microbial composition of raw mare milk, like that in other similarly less common milk types, has not been studied in depth: nevertheless, it is known to be widely variable, depending on many factors, such as the breed, season, and region [34]. However, many studies regarding the microbiological composition of fermented mare milk are available.

Koumiss, airag and chigee are the main products obtained through mare milk fermentation. Mare milk fermentation is due to lactic acid bacteria (LAB) and yeast interaction. LAB are mainly involved in milk acidification, whereas the yeasts partly modify its titratable acidity due to the production of acidic compounds, such as acetic acid. On the other hand, yeasts produce ethanol that is very important for determining the properties and increasing the stability of fermented milk [21,35].

The interaction between LAB and yeast can be complex and has to be studied in more depth; however, different theories have been suggested. Positive relationships between the two types of microorganisms can occur because lactic acid bacteria are responsible for the lowering of the pH due to the secretion of organic acids, allowing the yeast population to become competitive in the immediate environment, followed by yeast fermentation [36]. Sudun et al. observed a positive correlation regarding glucose and galactose produced by the lactase of LAB, and consumed by yeast for their growth [21].

In addition, the interaction between the two types of microorganisms is reflected in the product. Regarding its safety, the combination of the acidic condition, saturated with carbon dioxide and alcohol, is inhibitory to many spoilage bacteria [36]. Regarding quality, yeast proteolytic and lypolitic metabolism can stimulate LAB growth and play an important role in aroma development [37].

Koumiss is a lactic acid–alcoholic beverage produced traditionally by the fermentation of mare’s milk by indigenous organisms or by a starter culture. First, lactic acid is formed, and then alcoholic fermentation of the residual sugar content occurs. Koumiss generally contains about 2% alcohol, 0.5–1.5% lactic acid, 2–4% sugar and 2% fat (Kerr and McHale [38]; quoted by Danova et al. [39]). The alcohol has been known to reach a level of 3.5% and, depending on its content, in Russia there is a differentiation between weak koumiss with 0.7–1% ethanol, normal koumiss with 1–1.75% and strong koumiss with 1.75–2.5% [40].

According to Danova et al., another distinction exists: depending on the lactic acid content, three types of koumiss can be distinguished: ‘strong’, ‘moderate’ and ‘light’ koumiss. Lactic acid bacteria (*Lactobacillus delbrueckii* subsp. *bulgaricus* and *Lactobacillus rhamnosus*) acidifying the milk to pH 3.6–3.3 and converting about 80–90% of lactose into lactic acid produce ‘strong’ koumiss. In ‘moderate’ koumiss, other *Lactobacillus* (*Lb.*) bacteria (*Lb. acidophilus*, *Lb. plantarum*, *Lb. casei*, and *Lb. fermentum*) with lesser acidification properties and a conversion ratio of about 50% lower the pH to 4.5–3.9 at the end of the process. ‘Light’ koumiss, produced by *Streptococcus thermophilus* and *Str. cremoris*, is slightly acidic (pH 4.5–5.0) [39]. According to several authors, “moderate” koumiss presents a sweet–sour taste and a yeasty odour and is the most appreciated [39,41]. It was demonstrated that acetaldehyde is the most important substance determining aroma in koumiss and the suitable range of its content is 78.25−257.07 μL L^−1^ [42,43].

Airag, which is called also tsege in Inner Mongolia, contains *Lb. helveticus*, *Lactobacillus fermentum*, and *Saccharomyces cerevisiae* [44]. The traditional technique of production in a cowhide vessel of this product in Mongolia was included in 2019 in the Representative List of the Intangible Cultural Heritage of Humanity.

In an attempt to identify which properties are related to the most appreciated sensory characteristics of airag, it was found that only its electrical conductivity has a statistically significant relationship with its taste score: higher electrical conductivity values are associated with lower taste scores; even though the mean pH values were not different, calcium and phosphorus concentrations were lower in the airag samples rated with high scores, taking in account 51 different airag samples exhibited in a competition in Mongolia [22].

Many mesophilic LAB and yeasts have been detected in koumiss. According to tradition, fresh milk is inoculated with a small quantity of already-fermented milk as natural starter, but many other different substances could be used as well [19]. The use of raw milk and natural undefined starter cultures causes a strong variability in the microbial composition. Many authors have observed a wide variability in species and strains [27,45]. It has been evidenced that milk derivatives from each family have their particular microbiota [46].

A few studies have investigated the relationship between koumiss microbiota and the production of volatile flavour compounds or organic acids, and their effects on taste. Despite the wide variability of microbes and yeasts involved, a “core” microbiota was identified, represented by four bacterial *genera* (*Lactobacillus*, *Acetobacter*, *Lactococcus*, and *Pseudomonas*), and two yeast *genera* (*Kazachstania* and *Candida*), and it was observed that notwithstanding the differences depending on the region and production techniques, the basic volatile flavours in traditional koumiss are similar [45,47].

Since this product is administered in hospitals, in the former USSR guidelines were started in 1969 to regulate its production according to a standard: in this case, a pure culture should be used as starter (with *L. bulgaricus* and *S. lactis*, which have antibacterial properties). Regardless, according to soviet authors, afterwards old koumiss was commonly used as starter [19].

Like in other fermented dairy products, the genus *Lactobacillus* (*Lb.*) plays an important role in affecting the aroma, texture, and acidity of koumiss. Recently, much interest has been focused on the usage and safety of these strains, as the properties of probiotics are more known and appreciated. For this reason, special attention has been paid to the accurate identification and characterization of a potential probiotic microorganism to use as the selected starter. Regarding probiotics selection, it is necessary to assess properties affecting specific health benefits, such as the modulation of the immune system, survival and persistence in the host, and proven safety and stability. Regarding LAB identification, the comparison of molecular sequences, mainly 16S rRNA-encoding genes, is commonly used even if it is not always effective to identify genetically close species. It was stated that if the 16S rRNA gene sequence identity shared by two microorganism is lower than 97%, at the genomic level they are considered to belong to different species. If the shared identity values are higher than 97% or the sequences are identical, the organisms appear closely related and total DNA–DNA hybridization data or more discriminative analyses are needed for species identification [48].

Regarding LAB characterization, several techniques can be used, from the more traditional phenotypic approach like whole-cell protein and cell wall composition analysis, and other morphological, physiological, and biochemical analyses [49] to more recent molecular and genomic characterization [50]. Methods for characterizing probiotics were recommended in the advisory report of the Working Group “8651 Probiotics” of the Belgian Superior Health Council (SHC) by Huys et al. [51].

### 2.1. Lactic Acid Bacteria

Studies regarding the isolation, identification, and characterization of lactic acid bacteria (LAB) in fermented mare milk have been carried out mainly by Asian research groups in the regions where this production has been a tradition for centuries, namely, Mongolia and Inner Mongolia and the autonomic Region of China.

These kinds of studies began with a notable frequency in the early years of the 2000s, and are still actively going on, probably with the intent to find the most suitable combination of LAB for the industrial production of horse milk derivatives.

The main LAB genera and species characterized in recent years are summarized in Table 1. Different methodological approaches were used, also in consideration of the advent of new increasingly powerful methods.

Most of the knowledge of LAB in fermented mare milk has been gained through culture-dependent methods, and the subsequent isolation and identification of these microorganisms. The number of isolated and identified strains is strongly variable in the manuscripts, depending on the aim of each piece of research, varying from 2 for koumiss [63] to 258 for chigee [52]. In Table 1, the results of 18 studies carried out with several methods are shown. Briefly summarizing the species identified with these approaches, it is possible to conclude that 45 different species have been identified, but the most common were *Lb. plantarum*, identified in 12 of the 18 studies; *Lb. casei*, identified in 10 studies; *Lb. helveticus,* found in 9 studies; *Lb. kefiranofaciens,* found in 7 studies; and *Lb. paracasei,* found in 6 studies (Table 1). Less frequent were the species *Lb. fermentum*, *Lb. kefiri*, and *Leuconostoc mesenteroides,* found in 5 studies; *Lb. diolivorans,* found in 4 studies; *Lc. lactis* subsp. *lactis*, *Enterococcus faecium* and *S. thermophilus,* found in 3 studies; and *Lactococcus lactis* subsp. *cremoris*, *Lb. acidophilus*, *Lb. coryniformis*, *Lb. curvatus*, *Lb. farciminis*, *Lb. pentosus*, *Weissella kandleri* and *Lb. delbrueckii,* identified in two studies (Table 1). The other species were found only one time (Table 1).

Recently, considerable efforts have been made to develop more rapid, culture-independent methods. One study was carried out with this approach and in particular by means of denaturing gradient gel electrophoresis (DGGE) [64]. The authors concluded that the biodiversity of ten samples of collected koumiss, made by nomadic families in one region of China, was high. In particular, the dominant species identified by DGGE were *Lb. acidophilus*, *Lb. helveticus*, *Lb. fermentum*, and *Lb. kefiranofaciens*. Less frequent were *Enterococcus faecalis*, *Lactococcus lactis*, *Lb. paracasei*, *Lb. kitasatonis*, and *Lb. kefiri*. *Leuconostoc mesenteroides*, *Streptococcus thermophilus*, *Lb. buchneri*, and *Lb. jensenii* were occasionally found [64].

Comparing all the data, it is possible to highlight that the only species found with high frequency using the two approaches was *Lb. helveticus*, a thermophilic, homofermentative, proteolytic species traditionally used both for the manufacture of Swiss-type cheeses and long-ripened Italian cheeses or in the production of fermented beverages in north Europe [65]. However, due to its high proteolytic activity, *Lb. helveticus* is very effective in the production of bioactive peptides such as angiotensin-converting enzyme (ACE) inhibitory peptides [66]. The strain heterogeneity of this species isolated from koumiss was evidenced by an intra-species genotypic and phenotypic characterization [67].

The other three dominant species for DGGE have also been isolated and identified by culture-dependent research, but surprisingly, the species isolated in 12 of the 15 culture-dependent studies, *Lb. plantarum*, has not been recognized in the samples of koumiss analysed with DGGE. Even if this aspect suggests some considerations about the advantages and disadvantages of both approaches, other culture-independent research should be conducted to discuss this comparison.

More recently, new next-generation sequencing technologies have been applied to explore genes implicated in microbial metabolism: shotgun metagenomic analyses provide taxonomic and functional data about complex microbial communities, with culture-independent methods [68]. In particular, a study investigated bacterial function during several phases of koumiss fermentation by metagenomics. It was observed that the microbial composition of koumiss changes mostly in the first 36 h of fermentation, and the predominant species is *Lb. helveticus* [68].

The predominance of this species was observed in another study, which analysed the metagenomes of 23 koumiss samples collected in several regions of China: sequences representing 216 different species were found and *Lb. helveticus* comprised 78.9% of the total sequences in koumiss, followed by *Lactobacillus kefiranofaciens* (6.0%) and *Lactococcus lactis* (4.2%) [69].

Analysing the bacterial metagenomes of koumiss from Mongolia and Inner Mongolia by the single-cell genomics technique allowed the identification of rare bacterial species never detected in koumiss before, such as *Lb. otakiensis* and *Streptococcus macedonicus*, both present in other fermented foods or dairy products [70].

Since new genetic methods provide more precise metrics to classify bacteria, the genus *Lactobacillus* was recently re-evaluated and new genus and species names were recently adopted [71]. Therefore, in the more recent studies, LAB names are in accordance with the new taxonomy rules (see note for Table 1) [61,62].

Technological properties and probiotic aptitudes have been considered for strains isolated from koumiss and airag. In particular, probiotic aptitude, such as bile tolerance and other preliminary tests, has been evaluated for strains of *Lb. casei*, *Lb. helveticus* and *Lb. plantarum* [56] and for different strains of *Lb. acidophilus* [72]. Different technological properties have been investigated in several species. The fermentation properties of four *Lb. casei* strains were studied by Xu et al. [73], and in *Lb. rhamnosus* and *Lb. paracasei* by Zuo et al. [74]. The latter authors were also able to verify the performances of those strains in cheese and yogurt manufacturing [74]. The effects of distinctive proteolytic activity on casein degradation have been studied for six *Lb. helveticus* strains isolated from home-made airag samples [75].

In airag collected in Mongolia, Watanabe et al. isolated two novel microorganisms belonging to the genus *Bifidobacterium*. Their phenotypic and genotypic characteristics demonstrated that the strains can be considered a single *Bifidobacterium* species never observed before, and its proposed name was *Bifidobacterium mongoliense* sp. nov. [76].

### 2.2. Yeasts

The yeasts in koumiss are the main aspect responsible for the presence of ethanol and carbon dioxide. The amount of ethanol in koumiss is slightly higher than in kefir because the amount of lactose in mare milk is higher than in cow milk [9]. However, not only are lactose-fermenting yeast species, belonging to the genus *Kluyveromices* (*K.*), mainly *K. marxianus* and *K. fragilis*, and *Candida* (*C.*) *kefir,* common in koumiss, but also non-lactose-fermenting species, such as *Saccharomyces unisporus* (*S.*), are found [77]. A study which explored the correlation between microflora and volatile flavour substances demonstrated a correlation with the *genus Candida* and ethanol, considered the most important alcohol flavour compound [47].

In the manufacture of koumiss, a considerable amount of free amino acids are produced by yeasts, ranging around 470–490 mg kg^−1^ [40,43].

Few studies are available about the yeast composition of fermented mare milk. To the authors’ knowledge, six studies have been conducted on koumiss [24,37,47,60,77,78], one on chigee [79], one on airag [46] and one on hurunge, which is a the starter culture for fermented traditional dairy products such as chigee [54]. To briefly summarise, 11 genera and 24 species were isolated but only non-lactose-fermenting species *S. cerevisiae* was isolated in all the studies, and lactose-fermenting *K. marxianus* was found in seven of the nine studies. Lactose-fermenting species *C. krusei* and non-lactose-fermenting yeasts such as *Pichia* (*P.*) *membranaefaciens*, *C. kefyr*, *C. valida*, *Dekkera anomala*, *Kazachstania unispora*, and *Issatchenkia orientalis* were found in two studies, and the other species, *C. buinensis*, *C. pararugosa*, *Geotrichum* sp., *K. wickerham*, *P. cactophila*, *P. deserticola*, *P. fermentans*, *P. manshurica*, *P. membranaefaciens*, *S. dairensis*, *S. servazzii*, *S. unisporus*, *Trichosporum asaii*, *Penicillium carneum*, *Clavispora lusitaniae* and *Torulaspora delbrueckii,* were found in only one study (Table 2).

Interestingly, a polyphasic approach was used in a complete and complex study, using a culture-independent and also culture-dependent method, to study yeasts present in koumiss sampled from three representative regions of China, Mongolia, Xin Jiang and Qing Hai [37]. Using 96 samples, 655 isolates and also DGGE, the authors were able to show how the yeast community in koumiss is complex and rich in different species [37].

Another research article showed that the prevalent yeast found at high altitudes in Kazakhstan was *S. unisporus*, which was different from lower zones, where lactose-fermenting yeasts are more common, mostly belonging to the *Kluyveromyces genus* [77]. The authors hypothesized that a high altitude could affect the LAB population composition during the preparation of koumiss, with a selection of LAB not metabolizing galactose, causing therefore an enrichment of this sugar and the prevalence of *C. buinensis,* which ferments galactose but not lactose, and of the *Saccharomyces genus* compared with *Kluyveromyces* [77].

In traditional koumiss from Inner Mongolia, Guo et al. identified 57 fungal species, also, among them the genera *Penicillium*, *Cladosporium* and *Aspergillus* were detected in all samples. These are filamentous fungi considered the cause of spoilage in dairy products. Therefore, the production process of traditional koumiss requires sanitation measures and a better control of environmental hygiene [60].

In conclusion, it could be observed that in comparison with fermented dairy products from other species, in mare milk the higher lactose content (6–7% compared to 4–5% of cow, yak, goat and camel’s milk) usually determines the prevalence of lactose-fermenting yeast strains, but this trait could be modified by the altitude effect, which, when operating in a selection of LAB, can increase the galactose content, causing therefore the major presence of galactose-fermenting yeast strains.

## 3. Potential Functional Properties of Microorganisms in Fermented Mare Milk for Human Consumption and Health

Probiotics are defined as ‘‘living microorganisms, which upon ingestion in certain numbers, exert health effects beyond inherent basic nutrition’’ [80]. Probiotic cultures in nutritional supplements, pharmaceuticals and functional foods are mainly constituted by LAB belonging to the genera *Lactobacillus* and *Bifidobacterium*. The proteolytic systems of LAB are exploited for the production of bioactive peptides in fermented dairy products, because milk proteins need to be hydrolysed into peptides to exert some effect [81].

It is believed, but not yet definitively proven, that probiotics compete with undesirable microorganisms and can inhibit their growth in the intestine. Therefore, probiotics must survive through the stomach and upper intestine, tolerating the acidic and protease-rich environment and the action of bile salts, and need to be numerous enough to exert their effect in the colon [82]. According to several authors, a concentration of at least 10^6^ c.f.u.g^−1^ of viable and active microorganisms is necessary in the product throughout its specified shelf life [83].

Many recent studies regard koumiss as a source of microorganisms with potential probiotic activities, and trials have been carried out to provide data about these properties [84].

**Figure 1:**
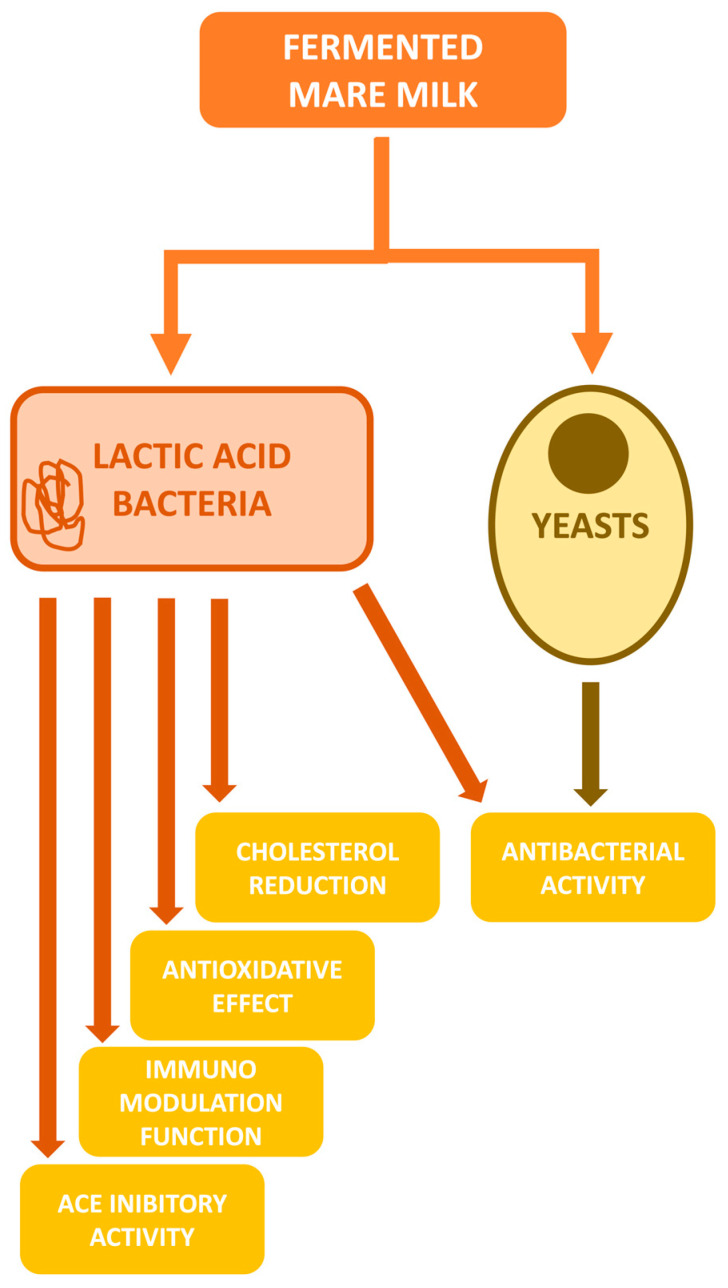
Main potential functional properties of microorganisms in fermented mare milk for human consumption and health.

In Figure 1, a synthesis of the main potential functional properties of microorganisms in fermented mare milk is shown.

A Chinese research group has been particularly active in this research field: during the years 2004–2009, 240 *Lactobacillus* strains were isolated from koumiss and investigated. Among these strains, a novel strain, called *Lb. casei* Zhang, was screened out and its potential probiotic properties were investigated in many trials, reported initially in national and successively in international journals. According to the combined analyses of a phylogenetic dendrogram and partial sequences of 16S rDNA, this strain was classified as *Lb. casei* subsp. *casei* [1,63,73]. The complete genome sequence of *Lb. casei* Zhang was successively investigated by a whole-genome shotgun strategy, and comparative analyses with other *Lb* strains evidenced a richer enzyme abundance, which could explain some of its favourable properties in terms of its use of different sugar sources and behaviour in the host environment [85]. Moreover, proteins expressed by *Lb. casei* Zhang in the exponential and stationary phases were identified and characterized, mainly as stress response proteins, in a proteomics study, evidencing their role in its adaptation to the environment [56]. Therefore, the survival capacity of this strain was initially tested: yoghurt samples fermented with *Lb. casei* Zhang showed a similar viable count (1.0 × 10^9^ c.f.u. mL^−1^) as other samples inoculated with selected commercial probiotics after 28 d of refrigerated storage [83]. Similar results were obtained by Zhou et al. [1].

In the following years, the properties of this strain were investigated with in vivo experiments on rats and on humans; some of the results of these studies, showing positive effects on several health problems, are listed by He et al. in the introduction of their paper, concerning the effects of the long-term administration of *Lb. casei* Zhang on human gut microbiota (see next section) [86].

Particular emphasis about the potential properties of the microorganisms from koumiss in the prevention of chronic diseases is shown in a recent review by Xue et al. [84].

The following sections consider the results of studies aimed at assessing several properties of LAB strains from koumiss as potential probiotics.

### 3.1. Survival of LAB through the Human Digestive Tract and Cholesterol Reduction Effect

The survival capacity of *Lactobacillus* strains isolated from mare milk products through the human digestive tract is considered in several studies. In particular, survival under acidic conditions, similar to gastric juice, and resistance to bile salts have been investigated. Bile salts have antibacterial action but do not damage resident microflora. The physiological concentration of human bile ranges from 0.3 to 0.5% (wt vol^−1^) [87]. It was recently observed that some *Lactobacillus* strains excrete bile salt hydrolase, an enzyme that catalyses the hydrolysis of conjugated bile salts. This action could reduce serum cholesterol levels, together with various other mechanisms [88]. According to Guo et al. [83], in vitro tests demonstrated *Lb. casei* Zhang’s tolerance to simulated gastric and intestinal juices and in the presence of 0.3% bile salts. The molecular mechanisms involved in the adaptation of the bacterial cells under bile salt stress were investigated considering the growth and protein expression patterns of *Lb. casei* Zhang with and without bile salts. It was observed that twenty-six proteins were differentially expressed using two-dimensional gel electrophoresis. These proteins were identified by peptide mass fingerprinting [87].

In the empty human stomach, pH is lower than 3.0 [89]. The tolerance to a low pH of *Lb. casei* strains from home-made koumiss was investigated considering the expression of H+-ATPase, which is supposed to play an important role in the maintenance of physiological cytoplasmic pH, by means of a mechanism controlling the H^+^ concentration through the cell membrane. The survival of the *Lb. casei* Zhang strain in artificial digestion was observed and the expression of H^+^-ATPase, detected by the reverse transcription–polymerase chain reaction method, increased accordingly to the pH lowering, in accordance with what has been observed regarding other microorganisms. Therefore, it seems that the acid tolerance of *Lb. casei* could have some relationship with the H^+^-ATPase gene [89].

It is assumed that an LAB strain could tolerate gastric acid if, after exposure to pH 3.0 with 0.04% pepsin for 3 h, it is detected at over 7 log c.f.u. mL^−1^. *Lb. plantarum* 05AM23 isolated from Mongolian airag showed good bile acid tolerance (97% viable colonies), viability in low pH (8.1 log c.f.u. mL^−1^) and a high capacity of adhesion on Caco-2 cells (72.0 × 10^3^ c.f.u. mL^−1^) [58].

There is an increasing interest in the research of natural compounds in food with effective abilities to decrease serum cholesterol concentrations, especially in countries where coronary heart disease is the principal cause of death, such as China. It has been known for many years that several *Lactobacillus* strains exert this property by means of different mechanisms, mostly studied by in vitro experiments [90,91]. According to some studies, which were published in Chinese national journals, several *Lb* strains have been observed in diverse acidic conditions (pH 3.0 and pH 4.0) and bile salt concentrations (e.g., in media containing 0.6%, 0.4% and 0.3% bile salts) in vitro: some of them showed sufficient tolerance and the cholesterol removed from the growth media was around 50% [72,92]. These properties were confirmed for the strain *Lb. helveticus* MG2-1, which, moreover, showed a good adhesiveness to Caco-2 cells, an important property to be assessed as a potential probiotic [93].

Evaluation of the cholesterol-reducing effects of LAB has been performed in vivo as well by several studies; e.g., the acid and bile tolerance and cholesterol reduction activity of *Lb. fermentum* SM-7, isolated from home-made koumiss in Xinjiang, China, were observed in vitro and in vivo in artificially induced hyperlipidaemial ICR mice. The cholesterol reduction rate in vitro observed in this study was 66.8%, and a significant decrease in serum total cholesterol was also observed in vivo, in the groups treated with high and low doses of *Lb. fermentum* SM-7 [90].

Nevertheless, observing the behaviour of three different strains, screened out from 68 *Lb* strains from koumiss produced in the same area mentioned above, it was concluded that the results in vitro were not consistent with what was observed in vivo in mice, and a precise explanation of the mechanisms of the hypocholesterolemic action of LAB is seemingly quite difficult, also because different strains show different cholesterol-lowering actions [91].

Regarding this issue, Zhong et al. [94], carrying out one of the aforementioned numerous studies conducted in China into *Lb. casei* Zhang, investigated in rats how the expression of several genes involved in fatty acid metabolic processes was differently affected by the administration of *Lb. casei* Zhang, therefore providing some information about the molecular mechanism of the cholesterol-lowering effect exerted by this bacterium [94].

Another quite recent study demonstrated, by metagenomic and metatranscriptomic profiling, that in the human gut, the gene expression of *Lb. casei* Zhang was very different from what was observed in vitro. Individual variabilities in the intestinal microbiomes among the healthy volunteers were higher than those induced by the probiotic ingestion. Therefore, interactions between probiotics and resident microbiota are a further step in the study of the effects of these promising strains [95]. Moreover, gut microbiota vary along the different life phases, and the differences in the represented phyla in the elderly gut, in comparison with those of younger adults, are well known.

In relation to this issue, He et al. studied the effects of the long-term administration of this strain on the gut microbiota of healthy adults, divided into two groups according to age. Modulating and stabilizing effects of *Lb. casei* Zhang on gut microbiota were observed in both groups, but with age-dependent differences. According to some indices, the long-time consumption of *Lb. casei* Zhang changed the microbiota composition in the older adult group, making the microbial community more similar to that of younger people [86].

Another study [96] considered thirteen patients, diagnosed with severe hyperlipidaemia, who consumed 750 g of koumiss every day for 60 days. The composition of the koumiss microbiota and the effects of koumiss consumption on patients’ faecal sample microbiota were investigated by the Pacific Biosciences single-molecule, real-time sequencing technology (SMRT), a state-of-the-art tool which permits the profiling of microbiota. This technique produces long-read sequences, allowing high-resolution taxonomic identification by sequencing the full-length 16S rRNA gene. After 60 days of koumiss consumption, HDL cholesterol values increased significantly, and hyperlipidaemia-associated symptom scores decreased significantly. These results were attributed to the bacterial population of koumiss and its metabolites. According to several indices, participants’ faecal samples reflected a significant increase in the abundance and diversity of gut microbiota. This was the first study which observed the relationship between koumiss consumption and gut microbiota at the species level in hyperlipidaemic patients [96].

### 3.2. Antioxidative Effect

The action of binding toxins is among the favourable properties attributed to fermented mare milk and its microorganisms, operating like natural chelators to remove the pollutants from the body. This property and the antioxidative effect of koumiss and its LAB strains have been investigated in a rat model: the strain *Lb. acidophilus* MG2-1, heat-killed or living, extracted from koumiss, was orally administrated for 28 days and an antioxidative effect was observed in serum and in rat liver tissue homogenate. In rats fed living *Lb. acidophilus* MG2-1, the activity of superoxide dismutase (SOD) and of glutathione peroxidase (GSH-PX) significantly increased in liver tissue and in serum, while the content of malondialdehyde in serum and liver tissue homogenate decreased [97].

The oxidative stress effects and damage induced in the rats, which received 25 ppm mercury (HgCL_2_) in drinking water for 6 weeks, was alleviated in the rat group fed fermented mare’s milk prepared with starter cultures of *Streptococcus thermophilus*, *Lb. acidophilus* and *Bifidobacterium bifidum* and supplemented with fibre (6% extract of Dandelium root, *Taraxacum officinalis*). Nevertheless, the mechanism of action was not explained, and the authors declared that it was still under investigation [98].

### 3.3. Immuno-Modulation Function

Some studies have been performed to assess a potential immuno-modulating function of probiotic species isolated from koumiss [99]. Among them, five studies considered the effects of live or heat-killed probiotic *Lb. casei* Zhang administration on healthy or liver-injured rats or mice.

For example, varying doses of *Lb. casei* Zhang, isolated from koumiss, were orally administered to healthy BALB/c mice and positive effects on several parameters of immune response were observed, such as the increased production of interferon-γ (IFN-γ) and decreasing levels of tumour necrosis factor-α (TNF-α). Moreover, interleukin-2 (IL-2) and IL-2 receptor gene transcription increased, and the production of secretory Immunoglobulin A (sIgA) was enhanced. It was concluded that the dose-dependent administration of living *Lb. casei* Zhang influences immune responses in mice and could be taken into account for the probiotic’s use for humans as well [100,101].

The research group of Wang et al. also observed several protective actions of *Lb. casei* Zhang in rats with induced liver injury, and demonstrated a positive effect of *Lb. casei* Zhang upon pro-inflammatory cytokine and hepatic inflammation in rats with acute liver failure [102,103,104].

### 3.4. ACE Inhibitory Activity

Angiotensin I-converting enzyme (ACE; dipeptidyl carboxypeptidase, EC3.4.15.1) is a multifunctional enzyme which plays a role in the conversion of angiotensin-I to angiotensin-II and in the degradation of bradykinin, causing increased peripheral blood pressure [105]. It has been demonstrated that the proteolytic systems of several LAB produce bioactive peptides exerting an inhibitory activity on the ACE, with a hypotensive effect in the rat model and in clinical studies. In particular, *Lb. helveticus* cell-wall proteases can activate antihypertensive tripeptides from the hydrolysis of casein. This capacity is strain-dependent and the effect can be exerted by milk fermented by these strains [106,107].

In koumiss collected in the Xilingole region in Inner Mongolia, four ACE-inhibitory peptides, called P_I_, P_K_, P_M_ and P_P,_ were identified and analysed: P_I_ was derived from mare milk β-casein (f213–241), and the peptide P_K_ was derived from *Actinobacillus succinogenes*, which had not been previously found in koumiss, whereas the other two peptides did not correspond to any known milk peptides or proteins. The in vitro studies demonstrated the thermal stability of the four peptides and a high ACE-inhibitory activity, maintained under various pH and ACE treatments [18].

Another antihypertensive property of some LAB strains is the production of gamma-aminobutyric acid (GABA), a neurotransmitter which is effective in lowering blood pressure. Sixteen *Lactobacillus* strains isolated from koumiss collected in Xinjiang, China, demonstrated high ACE inhibitory activity, and among them two strains produced GABA: the *Lb.*–ND01 strain, which showed 99% homology to *Lb. helveticus* according to the sequence of 16S rDNA, possesses both properties, and after further studies regarding its resistance to acidic conditions and its production of free amino nitrogen, it was considered interesting for its possible employment in probiotic dairy products [106].

### 3.5. Antibacterial Activity of LAB and Yeasts

The growth of pathogenic bacteria could be inhibited by particular toxins, produced by LAB or yeasts present in several fermented foods.

Many LAB produce a high diversity of bacteriocins, proteinaceous or peptidic toxins categorized in several ways, which kill or inhibit the growth of similar bacterial strains. Antimicrobial activity was observed in as many as 53 LAB strains isolated from airag. Class II bacteriocins of LAB are considered to have a large potential for food preservation, due to their anti-*Listeria* activity and physicochemical properties [108,109]. Bacteriocins produced by LAB isolated from fermented mare’s milk were characterized and tested for their antibacterial effect against yeasts and spoilage bacteria. It was concluded that bacteriocins A5-11A and B, isolated from *Enterococcus durans* issued from airag, could be used in food preservation due to their anti-pathogenic-bacteria activity, whereas yeasts were not inhibited [110]. Another interesting bacteriocin was identified in airag from the strain *Leuc. mesenteroides* 406, which showed a narrow antimicrobial spectrum but is effective against *Listeria monocytogenes* and *Clostridium botulinum* [108].

Recently, it was demonstrated that a bacteriocin produced by *Lb. plantarum* NMD-17, isolated from koumiss from Inner Mongolia, has a broad spectrum against Gram-positive and Gram-negative bacteria [111], and a bacteriocin produced by *Lb. rhamnosus* 1.0320, also extracted from koumiss, besides its wide-spectrum action, is particularly active against *E. coli* UB1005 [112].

Whereas the antimicrobial properties of LAB are widely documented, less is known about similar properties in yeasts extracted from koumiss. Particular yeast strains produce toxins called mycocins: compounds extracted by *Saccharomyces cerevisiae* from koumiss exerted antibacterial activity on pathogenic *Escherichia coli* in in vitro tests [113], and crude extracts of mycocins from *K. marxianus* showed efficacy against *Escherichia coli* in mice in vivo as well [114].

## 4. Conclusions

Research about mare’s milk and derivatives for human consumption is very active especially in Asian countries. The identification and characterization of LAB and yeasts have been performed in many studies: a wide variability of species and strains have been observed. Even if fermented mare’s milk is a very ancient product, a lot of research is still needed: whereas the yeast flora are quite well known, a wide range of scientific activity remains to be carried out to clarify the interdependency and cooperation of its various microbial components, because in fermented mare milk products really complex population structures of yeasts, white moulds, lactic acid bacteria and acetic acid bacteria are present and interacting.

Studies are actively going on to assess the properties of different strains for their optimal employment in the production, at an industrial scale, of koumiss, in particular.

Trials in vitro and in vivo are confirming some of the beneficial effects and healing properties traditionally attributed to mare milk derivatives: some probiotic properties of LAB in fermented milk have been demonstrated, but more necessary experimental steps must be performed to clearly assess their efficacy.

## Figures and Tables

**Table 1:** Main LAB species isolated in different studies.

^1^	^2^	Main LAB Species	Identification Method	Product	Sampling Location	References
10	258	*Enterococcus faecium*, *Lactobacillus casei*, *Lactobacillus paracasei*, *Lactobacillus plantarum*, *Leuconostoc mesenteroides* subsp. *dextranicum*, *Streptococcus thermophilus*	Growth condition, gas production, ammonia production, sugar fermentation, hippurate hydrolysis	chigee	Inner Mongolia	Burentegusi et al., 2002 [52]
14	117	*Lactobacillus pentosus*, *Lactobacillus plantarum*, *Lactococcus lactis* subsp. *cremoris*	Chemotyping, sugar fermentation, 16S rDNA sequencing	chigee	Inner Mongolia (Silinguole, Wulanchabu)	An et al., 2004 [23]
5	30	*Lactobacillus acidophilus*, *Lactobacillus casei*, *Lactobacillus plantarum*	Not mentioned	koumiss	Mongolia	Menghe et al., 2004 [53]
21	80	*Lactobacillus acidophilus*, *Lactobacillus casei*, *Lactobacillus coryniformis*, *Lactobacillus curvatus*, *Lactobacillus fermentum*, *Lactobacillus kefiranofaciens*, *Lactobacillus paracasei*, *Lactobacillus plantarum*, *Weissella kandleri*, *Weissella paramesenteroides*	Not mentioned	koumiss	Inner Mongolia	Menghe et al., 2004 [53]
2	7	*Lactobacillus buchneri*, *Lactobacillus plantarum*, *Lactobacillus salivarius*	Growth condition, gas production, ammonia production, sugar fermentation, hippurate hydrolysis	lyophilized koumiss	Mongolia	Danova et al., 2005 [39]
3	66	*Lactobacillus acetotolerans*, *Lactobacillus casei*, *Lactobacillus homohiochii*, *Lactobacillus kefiranofaciens*, *Lactobacillus plantarum*, *Lactococcus lactis* subsp. *cremoris*, *Lactococcus lactis* spp. *lactis*, *Lactococcus raffinolactis*, *Leuconostoc mesenteroides* subsp. *cremoris*	Growth condition, gas production, sugar fermentation	hurunge	Inner Mongolia	Shungquan et al., 2006 [54]
3		*Lactobacillus farciminis*, *Lactobacillus helveticus*, *Lactobacillus kefiri*, *Lactobacillus paracasei*, *Lactobacillus plantarum*	sugar fermentation, 16S rDNA sequencing	airag	Mongolia	Uchida et al., 2007 [46]
	12	*Lactobacillus casei*, *Lactobacillus fermentum*, *Lactobacillus helveticus*, *Lactobacillus plantarum*	Physiological tests and 16S rDNA sequencing	koumiss	China	Wang et al., 2008 [49]
22	183	*Enterococcus faecium*, *Lactobacillus casei*, *Lactobacillus diolivorans*, *Lactobacillus farciminis*, *Lactobacillus helveticus*, *Lactobacillus hilgardii*, *Lactobacillus kefiranofaciens*, *Lactobacillus kefiri*, *Lactobacillus parafarranginis*, *Lactobacillus plantarum*, *Lactococcus lactis* subsp. *lactis*, *Lactococcus* spp, *Leuconostoc mesenteroides*, *Leuconostoc pseudomesenteroides*, *Streptococcus thermophilus*	RAPD-PCR multiplex and 16S rDNA sequencing	airag	Mongolia	Watanabe et al., 2008 [55]
16	48	*Lactobacillus casei*, *Lactobacillus coryniformis*, *Lactobacillus curvatus*, *Lactobacillus fermentum*, *Lactobacillus helveticus*, *Lactobacillus kefiranofaciens*, *Lactobacillus paracasei*, *Lactobacillus plantarum*, *Weissella kandleri*	Growth condition, sugar fermentation, acid resistance, bile tolerance, 16S rDNA sequencing	koumiss	Inner Mongolia	Wu et al., 2009 [56]
5	26	*Lactobacillus casei*, *Lactobacillus helveticus*, *Lactobacillus plantarum*	Growth condition, gas production, ammonia production, sugar fermentation, lactic acid isomers, 16S rRNA sequencing	airag	Mongolia	Sun et al., 2010 [57]
7	7	*Lactobacillus delbrueckii* subsp. *Lactis*, *Lactobacillus fermentum*, *Lactobacillus helveticus*, *Lactobacillus kefiri*, *Lactobacillus pentosus*, *Lactobacillus plantarum*, *Lactobacillus sakei*, *Lactococcus lactis* subsp. *lactis*, *Leuconostoc citreum*, *Leuconostoc garlicum*, *Weissella confusa*, *Weissella viridescens*	16S rDNA sequencing, acid resistance, bile tolerance, adhesion to Caco2 cells, RAPD-PCR	airag	Mongolia	Takeda et al., 2011 [58]
	18	*Lactobacillus helveticus*, *Lactobacillus kefiranofaciens*, *Lactobacillus kefiri*, *Lactobacillus diolivorans*, *Enterococcus faecium*, *Enterococcus durans*	Growth condition, sugar fermentation, acid resistance, bile tolerance 16S rDNA sequencing BLAST search program	airag	Mongolia	Choi, 2016 [59]
11		*Lactococcus lactis*, *Lactobacillus buchneri*, *Enterococcus italicus*, *Lactobacillus homohiochii*; *Lactobacillus hilgardii*, *Lactobacillus helveticus*, *Leuconostoc mesenteroides*, *Streptococcus parauberis*	16S rDNA sequencing RAPD-PCR	koumiss	Inner Mongolia	Guo et al., 2019 [60]
15	109	*Lacticaseibacillus paracasei*, *Limosilactobacillus fermentum*, *Lacticaseibacillus casei*, *Lentilactobacillus diolivorans*, *Lactobacillus helveticus*, *Schleiferilactobacillus harbinensis*, *Leuconostoc mesenteroides*, *Lactobacillus kefiranofaciens*, *Lentilactobacillus parabuchneri*, *Staphylococcus epidermidis* ^3^	16S rRNA gene sequencing, PacBio SMRT sequencing technology	koumiss	Kyrgyzstan	Sun et al., 2022 [61]
15	26	*Lactobacillus helveticus*, *Lactobacillus delbrueckii*, *Lactobacillus kefiranofaciens*, *Lentilactobacillus diolivorans*, *Lentilactobacillus kefiri* ^3^	16S rRNA gene sequencing, PacBio SMRT sequencing technology	koumiss	Uzbekistan	Sun et al., 2022 [61]
14		*Lacticaseibacillus paracasei*, *Lacticasebacillus casei*, *Lacticaseibacillus rhamnosus*, *Lactobacillus delbrueckii*, *Lactobacillus delbrueckii* subsp. *bulgaricus*, *Enterococcus faecalis*, *Streptococcus thermophilus* ^3^	16S rRNA gene sequencing	koumiss	Kazakhstan (Almaty and Zhambyl regions)	Oleinikova et al., 2024 [62]

^1^ Number of samples; ^2^ Number of isolates; ^3^ In the studies after 2022, a new nomenclature was adopted for LAB. See http://lactobacillus.ualberta.ca/, (accessed on 19 December 2023).

**Table 2:** Main yeast species isolated from fermented mare milk in different studies.

^1^	^2^	Main Yeast Species	Identification Method	Product	Sampling Location	References
94	417	*Candida buinensis*, *Kluyveromyces marxianus*, *Saccharomyces cerevisiae*, *Saccharomyces unisporus*	morphological and physiological tests	koumiss	Kazakhstan	Montanari et al., 1996 [77]
3	nd	*Kulyveromyces wickerham*, *Issatchenkia orientalis*, *Saccharomyces cerevisiae*, *Saccharomyces dairensis*	sugar fermentation, 26S rDNA sequencing	airag	Mongolia	Uchida et al., 2007 [46]
96	655	*Candida pararugosa*, *Dekkera anomala*, *Geotrichum sp.*, *Issatchenkia orientalis*, *Kazachstania unispora*, *Kluyveromyces marxianus*, *Pichia deserticola*, *Pichia fermentans*, *Pichia manshurica*, *Pichia membranaefaciens*, *Saccharomyces cerevisiae*, *Torulaspora delbrueckii*	5.8S-ITS rDNA and 26S rDNA	koumiss	Mongolia, Xin Jiang, Qing Hai	Mu et al., 2012 [37]
5	108	*Candida kefyr*, *Candida krusei*, *Candida valida*, *Kluyveromyces marxianus*, *Pichia cactophila*, *Saccharomyces cerevisiae*, *Saccharomyces servazzii*	API ID and physiological tests	chigee	Mongolia	Sudun et al., 2010 [79]
28	87	*Kluyveromyces marxianus*, *Pichia membranaefaciens*, *Saccharomyces cerevisiae*, *Saccharomyces unisporus*	biochemical tests, 26S rDNA sequencing	koumiss	Xin Jiang	Ni et al., 2007 [24]
3	30	*Candida kefyr*, *Candida krusei*, *Candida valida*, *Kluyveromyces marxianus*, *Saccharomyces cerevisiae*	API ID and physiological tests	hurunge	Inner Mongolia	Shungquan et al., 2006 [54]
11		*Kluyveromyces marxianus*, *Kazachstania unispora*, *Dekkera anomala*, *Saccharomyces cerevisiae*, *Trichosporum asaii*, *Penicillium carneum*, *Pichia membranaefaciens*, *Clavispora lusitaniae*	ITS rDNA, 16S rDNA sequencing	koumiss	Inner Mongolia	Guo et al., 2019 [60]

^1^ Number of samples; ^2^ number of isolates.

## Data Availability

Data are contained within the article.
